# A novel deep-learning based weighted feature fusion architecture for precise classification of pressure injury

**DOI:** 10.3389/fphys.2024.1304829

**Published:** 2024-02-22

**Authors:** Dongfang Wang, Lirui Guo, Juan Zhong, Huodan Yu, Yadi Tang, Li Peng, Qiuni Cai, Yangzhi Qi, Dong Zhang, Puxuan Lin

**Affiliations:** ^1^ Department of Neurosurgery, Wuhan University Renmin Hospital, Wuhan, China; ^2^ School of Physics and Technology, Wuhan University, Wuhan, China; ^3^ Union Hospital Tongji Medical College, Huazhong University of Science and Technology, Wuhan, China; ^4^ Neurosurgery Department, Zhongshan Hospital Xiamen University, Xiamen, China

**Keywords:** pressure injury, deep-learning, fine-grained classification, weighted feature pyramid network, classification

## Abstract

**Introduction:** Precise classification has an important role in treatment of pressure injury (PI), while current machine-learning or deeplearning based methods of PI classification remain low accuracy.

**Methods:** In this study, we developed a deeplearning based weighted feature fusion architecture for fine-grained classification, which combines a top-down and bottom-up pathway to fuse high-level semantic information and low-level detail representation. We validated it in our established database that consist of 1,519 images from multi-center clinical cohorts. ResNeXt was set as the backbone network.

**Results:** We increased the accuracy of stage 3 PI from 60.3% to 76.2% by adding weighted feature pyramid network (wFPN). The accuracy for stage 1, 2, 4 PI were 0.870, 0.788, and 0.845 respectively. We found the overall accuracy, precision, recall, and F1-score of our network were 0.815, 0.808, 0.816, and 0.811 respectively. The area under the receiver operating characteristic curve was 0.940.

**Conclusions:** Compared with current reported study, our network significantly increased the overall accuracy from 75% to 81.5% and showed great performance in predicting each stage. Upon further validation, our study will pave the path to the clinical application of our network in PI management.

## 1 Introduction

Pressure injury (PI) refers to localized damage to the skin and soft tissue located over the elevations and is commonly found in bedridden patients. PI has a predilection for occurring in the skin and subcutaneous tissues above bony prominences, including the sacrococcygeal region and the heel region (Hajhosseini et al., 2020). In the United States, PI affects 3 million people each year, whereas in Europe, the incidence of hospital stress injury is approximately 8.3%–23% in different countries (Mervis and Phillips, 2019). With the prevalence of concomitant PI increasing year by year in the past decade, PI has become a global health problem. PI occurring during hospitalization is referred to as hospital acquired pressure injury (HAPI), and HAPI increases not only care time and treatment costs, but also family and social burdens (Gaspar et al., 2019). In addition, studies have shown that about 28% of stress injuries occur in home patients, while nearly 25% of them die from infections caused by PI. Researchers have shown that tissue damage in areas of intact skin penetrates from the deep layer to the surface within 48 h, after 7 h to 10 days, further deteriorating to necrosis. Therefore, early detection and timely prevention are effective ways to reduce the incidence of PI(4).

Notably, based on the 4-stage classification pf PI, the 2019 guideline issued by the National Pressure Ulcer Advisory Panel (NPUAP) further proposed several concepts such as pressure injury of unclear stage, deep tissue pressure injury, extension of pressure injury, mucosal pressure injury, and equipment related pressure injury, which extremely complex the staging and grading of PI (Munoz et al., 2020). First, its diagnosis in the clinical practice is highly dependent on the subjective judgment of caregivers, which is not conducive to a homogenous management in the clinic (Mervis and Phillips, 2019; Kottner et al., 2020; Munoz et al., 2022). Second, in post-charged patients, the care or family mostly has not received systematic medical training and it is difficult to make precise judgment about the progress and staging of PI, which on the one hand is prone to aggravating the condition and delaying diagnosis, and on the other hand, it is prone to increase the demand of patients’ hospitalization, resulting in the waste of medical resources. Therefore, there is a great need to construct more intelligent and precise PI grade quantification strategies, develop PI precision staging platforms, and provide theoretical guidance and therapeutic evidence for the care of PI.

To date, with the rapid advances in artificial intelligence, mining and parsing of medical images have entered a new stage. Digital quantitative high-throughput analysis is achieved by applying a large number of automated data characterization algorithms to transform image data of regions of interest (ROI) into feature space data with high resolution (Esteva et al., 2019). Automated analysis of PI images from patients in hospital or post-charged using artificial intelligence and giving corresponding treatment opinions to patients and families based on the results can greatly shorten treatment processes and save medical costs. We notice that there are parts of research teams have done some inspiring work in this area (Alderden et al., 2018; Jiang et al., 2020; Cai et al., 2021; Song et al., 2021; Jiang et al., 2022; Chen et al., 2023). The current studies primarily revolve around two aspects: (Hajhosseini et al., 2020): Creating a pressure injury dataset: they select diagnosed records of patients with pressure injuries from the cases of hospitalized patients in a medical center over the past few years. Early-stage images of the ulcer areas are obtained, and the core regions are selected, cropped, and resized to specific sizes. These prepared images are then handed over to professional doctors and nurses to classify and label them according to an international standard for the classification of pressure injuries. (Mervis and Phillips, 2019). Testing on the dataset with convolutional neural networks: the prepared dataset is divided into certain proportions and used to train on the state-of-art CNN architectures in order to find the network with the best classification performance for practical application.

The above-mentioned work does provide some assistance in PI care tasks. However, the scale of the PI dataset varies depending on the researchers’ data acquisition channels. From our research findings, the number of images ranges from a hundred to two thousand. If a multi-classification task is performed on a small dataset, the reliability and generalizability of the training results may be compromised. On the other hand, different from conventional classification tasks, subcategory images of pressure injuries exhibit closer similarities because the damaged areas all belong to the skin tissue, sharing many common features, which is a significant factor limiting the improvement of classification network accuracy (Ding et al., 2021). For instance, distinguishing stage 3 PI from others, both of which involve skin breakdown and exposure of the dermis, requires more detailed texture features for auxiliary judgment such as whether fat and deep tissues are exposed and whether there is granulation tissue, necrosis, or eschar. In the design of CNNs, deeper networks have larger receptive fields, allowing them to learn more semantic features. However, this inevitably leads to the loss of detailed information about small discriminative regions and these lower-level details such as texture patterns, colors, and edge connections, which are crucial for the classification of pressure injuries.

To address the above-mentioned issues, the main contributions of this study can be summarized as follows:

• Firstly, we established a large scale dataset consisting of 1,519 pressure injury images from multi-center cohorts which were classified by professional doctors and nurses according to NPUAP standards.
• Secondly, we test the performance of the latest state-of-art CNN architectures on our self-built dataset and compare the classification results using common evaluation metrics.
• Finally, based on the model with the highest classification accuracy, we propose a novel weighted feature fusion architecture, which combines a top-down pathway and a bottom-up pathway to fuse learned high-level semantic and low-level detailed representations for fine-grained classification. The dule-path structure fuses high-level semantic information and low-level detail representation, which can better differentiate pressure ulcer images with closer similarities. Meanwhile, the introduction of weights before fusion will correct the prediction results according to the importance of different scale feature layers, and finally achieve more accurate pressure ulcer classification results.

## 2 Methods

### 2.1 Dataset and exclusion

Our dataset consisted of retrospectively collected data that formed a convenience series. It contains 3 in-house datasets and 1 publicly available dataset. In-house datasets (Pressure Injury Image Dataset of Wuhan University Renmin Hospital, whu-PIID) were collected from 3 different institutes: Wuhan University Renmin Hospital [containing 363 images], Union Hospital Tongji Medical College Huazhong University of Science and Technology [containing 80 images], and Zhongshan Hospital Xiamen University [containing 76 images]. Public dataset (containing 1,091 image) were collected from the Pressure Injury Images Dataset (PIID) (Ay et al., 2022). The collected images were identified by experienced doctors and nurses (>10 years of experience).

After screening, patients with unstagable PI, suspected deep tissue damage, and mucosal PI were excluded. Their conditions were identified by experienced doctors and nurses. Finally, 291 images from Wuhan University Renmin Hospital, 72 images from Union Hospital Tongji Medical College Huazhong University of Science and Technology, 65 images from Zhongshan Hospital Xiamen University, and 1,091 images from PIID (a total of 1,519) were included.

### 2.2 Weighted feature pyramid network

**FIGURE 1:**
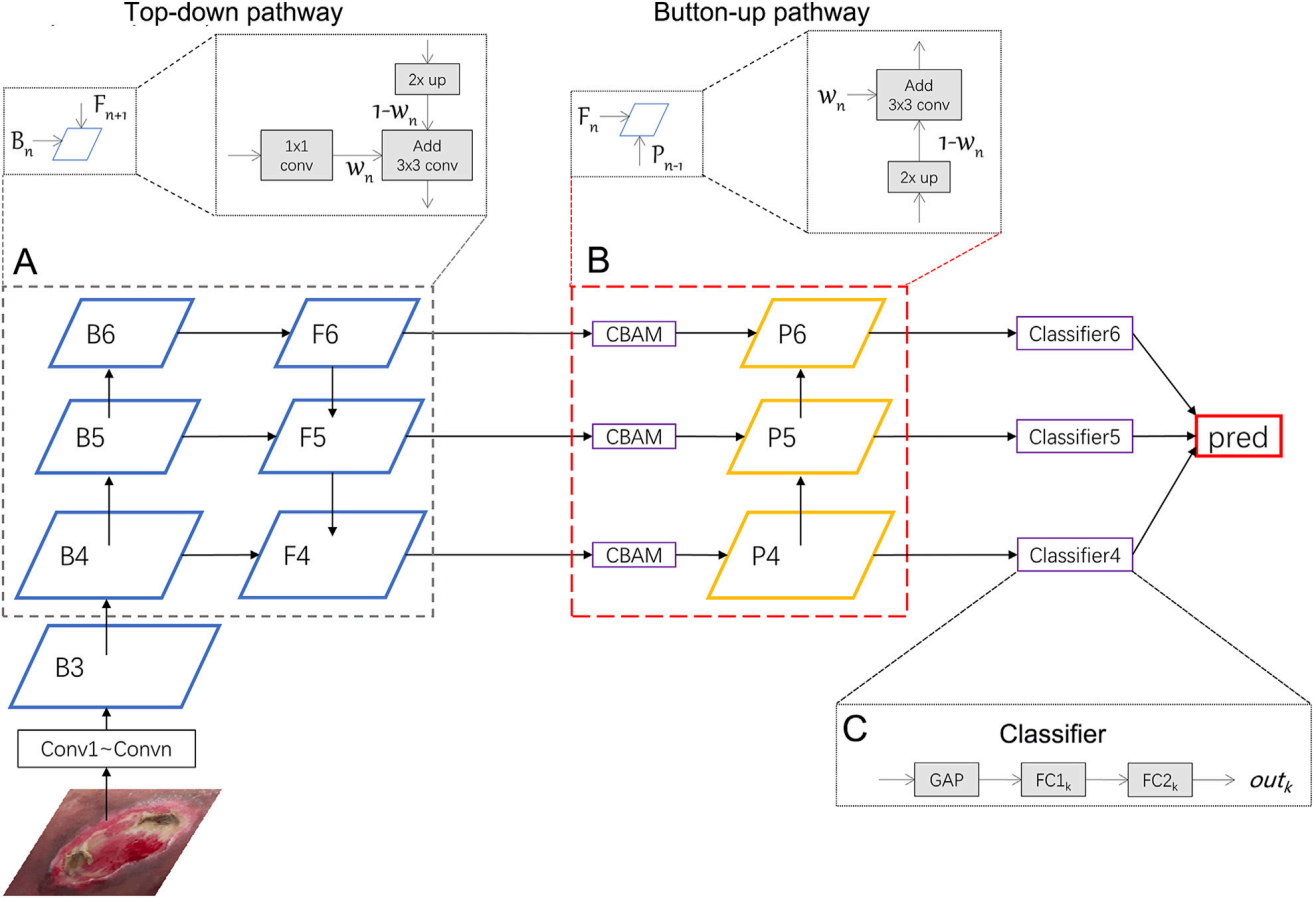
Illustration of our network. We uses ResNeXt as the backbone network and wFPN as the feature fusion network. In this figure, wFPN contains top-down **(A)** and bottom-up **(B)** weighted feature fusion pathways as shown in the lower dotted box, and the details were presented in the upper dotted box. The final result is obtained by weighting the outputs of the 3 classifiers **(C)**.

In this section, we will introduce the Weighted Feature Pyramid Network (wFPN) designed for pressure injury classification (Figure 1). We establish a dual-path feature fusion structure which combines top-down as well as bottom-up pathways and add an additional adaptive trainable weight to each input feature layer so that the network can adjust the importance of different input features by training.

1) ***Overview*** *:* The image input goes through the CNN backbones and obtains feature hierarchy {*B*_*1*_ *, B*_*2*_ *, B*_*3*_ *, …, B*_*6*_} through a series of convolutional blocks, where *n* represents the number of convolutional blocks. Typical image classification methods directly take the last feature map and connect it to a fully connected layer for classification. While the last feature map contains strong semantic information, it is weak in detailed representation of image features, which may result in unsatisfactory performance in fine-grained tasks where the differences between categories are small, such as pressure injury classification.

To address this issue, the FPN (Feature Pyramid Network) structure combines multi-scale feature maps through a top-down pathway, mapping high-level semantic information to low-level features and generating *l* corresponding feature hierarchy { $F_{n-l+1}$ , $F_{n-l+2}$ ,…, $F_{n-1}$ , $F_{n}$ }. Additionally, PANet (Path Aggregation Network) adds a top-up pathway to generate feature hierarchy { $P_{n-l+1}$ , $P_{n-l+2}$ ,…, $P_{n-1}$ , $P_{n}$ } to enhance the localization information of high level feature maps.

We further enhance the dual-path feature fusion network by introducing adaptive and learnable weight parameters for each feature map. We believe that the contribution of each feature map to the fused result should not be equally distributed when fusing features of different scales. Therefore, it is necessary to add the weight parameters for reaching more optimal results.

2) ***Weighted Feature Fusion***: Before fusing features, it is necessary to adjust the feature maps of different scales to the same resolution through methods such as up-sampling or down-sampling. Common weighting methods include softmax-based and fast normalization-based methods. Since softmax-based method involves exponential computations, which introduces additional computational complexity, we adopt the fast normalization method for feature fusion. The calculations are shown in the formula below:

$$O=\sum_{i}\frac{w_{i}}{\sum_{j}w_{j}+\epsilon }∙I_{i}$$ (1)

where $I_{i}$ represents the feature layer before fusion, $w_{i}$ is a nonnegative value representing the weights before normalization, and ϵ=0.0001 is a small value to prevent division by zero.

3) ***Dual Pathway Hierarchy***: We first select the last 3 layers {*B*_*4*_ *, B*_*5*_ *, B*_*6*_} and put them into the top-down pathway with weights. The process of obtaining $F_{n}$ from feature map $B_{n}$ can be shown as:

$$F_{n}=\frac{Conv3\left(w_{1}\times Conv1\left(B_{n}\right)+w_{2}\times Resize\left(F_{n+1}\right)\right)}{w_{1}+w_{2}+\epsilon },$$ (2)

where *conv1*() represents a 1 × 1 convolutional layer that adjusts the channel of different input features to the same size, *conv3*() represents a 3 × 3 convolutional layer that smooths the feature layer after adding the two feature layers. *Resize*() represents up-sampling to match the resolution of the fused feature layers, $w_{1}$ and $w_{2}$ are the weights assigned to the feature layer, controlling its contribution to the final output.

In particular, since the last feature map *B*_*6*_ does not have upper layers to be fused with, we perform global average pooling and max pooling on it to obtain the output for the next stage:

$$F_{6}=Conv1\left(AvgPool\left(B_{6}\right)+MaxPool\left(B_{6}\right)\right),$$ (3)

where *AvgPool*() represents global average pooling, *MaxPool*() represents global max pooling, and *conv1*() is used to adjust the channel size.

After the top-down pathway, we add a Convolutional Block Attention Module (CBAM) (Wang et al., 2022), which is an attention mechanism module that combines both channel and spatial attention to enhance the network’s perception of important regions in the image. In this module, the input feature map F first goes through the channel attention module and then the spatial attention module to obtain the refined final output feature. The process of channel attention module generating the feature map $F^{\prime }$ can be represented as:

$$F^{\prime }=M_{c}\left(F\right)⊗F$$ (4)

$$M_{c}\left(F\right)=\sigma \left(MLP\left(AvgPool\left(F\right)\right)+MLP\left(MaxPool\left(F\right)\right)\right),$$ (5)

where ⊗ represents element-wise multiplication, and broadcasting is used for dimension transformation and matching. *MLP*() represents a multi-layer perceptron that generates a 1D channel attention feature map, and σ represents the sigmoid function. The feature map $F^{\prime }$ goes through the spatial attention module to generate the final output $F^{\prime \prime }$ :

$$F^{\prime \prime }=M_{s}\left(F^{\prime }\right)⊗F^{\prime }$$ (6)

$$M_{s}\left(F^{\prime }\right)=\sigma \left(f^{7\times 7}\left(AvgPool\left(F^{\prime }\right);\;MaxPool\left(F^{\prime }\right)\right)\right),$$ (7)

where $f^{7\times 7}$ represents a convolution operation with a kernel size of 7, used to generate the 2D spatial attention feature map.

After the CBAM module, we also add a bottom-up pathway. Since the channel sizes of previous layers have been adjusted to 256, a 1 × 1 convolution have been removed. The process of obtaining $P_{n}$ from feature map $F_{n}^{\prime \prime }$ can be represented by the following equation:

$$P_{n}=\frac{Conv3\left(w_{1}\times F_{n}^{\prime \prime }+w_{2}\times Resize\left(P_{n-1}\right)\right)}{w_{1}+w_{2}+\epsilon },$$ (8)

here *Resize*() represents down-sampling.

4) ***Classifier***: The outputs {*out*_*4*_ *, out*_*5*_ *, out*_*6*_} are obtained from the classifiers after {*P*_*4*_ *, P*_*5*_ *, P*_*6*_} passing through a classifier that includes a global average pooling layer and two fully connected layers. The final prediction result is obtained by averaging the weighted sum of the three outputs:

$$pred=\frac{\sum_{k}w_{k}\times {out}_{k}}{\epsilon +\sum_{k}w_{k}}$$ (9)

### 2.3 Performance evaluation metrics

In order to objectively evaluate the multi-classification performance of various models on the pressure injury dataset, we employ parameters such as Confusion Matrix, Precision, Recall, Accuracy, and F1-score as evaluation metrics. The Confusion Matrix is a numerical table that summarizes the records in the dataset according to the true class and the predicted class of the classification model, providing an intuitive view of the classification results of the deep learning model for each class. Based on the Confusion Matrix, we can calculate true positive (TP), true negative (TN), false positive (FP), and false negative (FN). TP represents the number of positive samples correctly identified by the classifier, TN represents the number of negative samples correctly identified by the classifier, FP represents the number of negative samples incorrectly identified as positive, and FN represents the number of positive samples missed by the classifier. Using these four metrics, we can calculate Precision, Recall, Accuracy, and F1-score as shown below:

$$precision=\frac{TP}{TP+FP}$$ (10)

$$recall=\frac{TP}{TP+FN}$$ (11)

$$accuracy=\frac{TP+TN}{TP+FN+TN+FP}$$ (12)

$$F_{1-score}=\frac{2\times precision\times recall}{precision+recall}$$ (13)

Since this study involves multi-classification tasks, we use the macro F1-score to determine whether the images are evenly classified. Its calculation method is:

$${macro-F}_{1}=\frac{1}{n}\sum_{i=1}^{n}F_{i}$$ (14)

where $F_{i}$ represents F1-score of each category.

### 2.4 Experimental platform and details

The platform for this study is as follows: CPU: Intel Xeon E5-1660 (3.7 GHz); GPU: NVIDIA GeForce RTX 3060 (12 GB); DDR3: 64GB; Python 3.8.13, CUDA 11.3.

Before this experiment, we fix the random seed and randomly divide the dataset into a training set and a validation set in an 8:2 ratio. The total number of images at each stage and their distribution for training and validation are shown in Table 1. The mean and standard deviation of all images in the dataset are calculated to be [0.537, 0.424, 0.393] and [0.523, 0.416, 0.376] respectively. During the image preprocessing stage, the data is normalized using these values.

**TABLE 1:** The total number of images in training and validation set.

	Train	Validation	Total
Stage 1	308	77	385
Stage 2	419	104	523
Stage 3	255	63	318
Stage 4	235	58	293
Total	1,216	303	1,519

For network training, the AdamW (Loshchilov and Hutter, 2017) optimizer was used for network optimization. The initial learning rate and weight decay are set to be 0.0002 and 0.0001 respectively. The learning rate is dynamically updated using the cosine annealing function. The input image size is 224 × 224, and the batch size is set to 16.

## 3 Results

### 3.1 Patient characteristics

**FIGURE 2:**
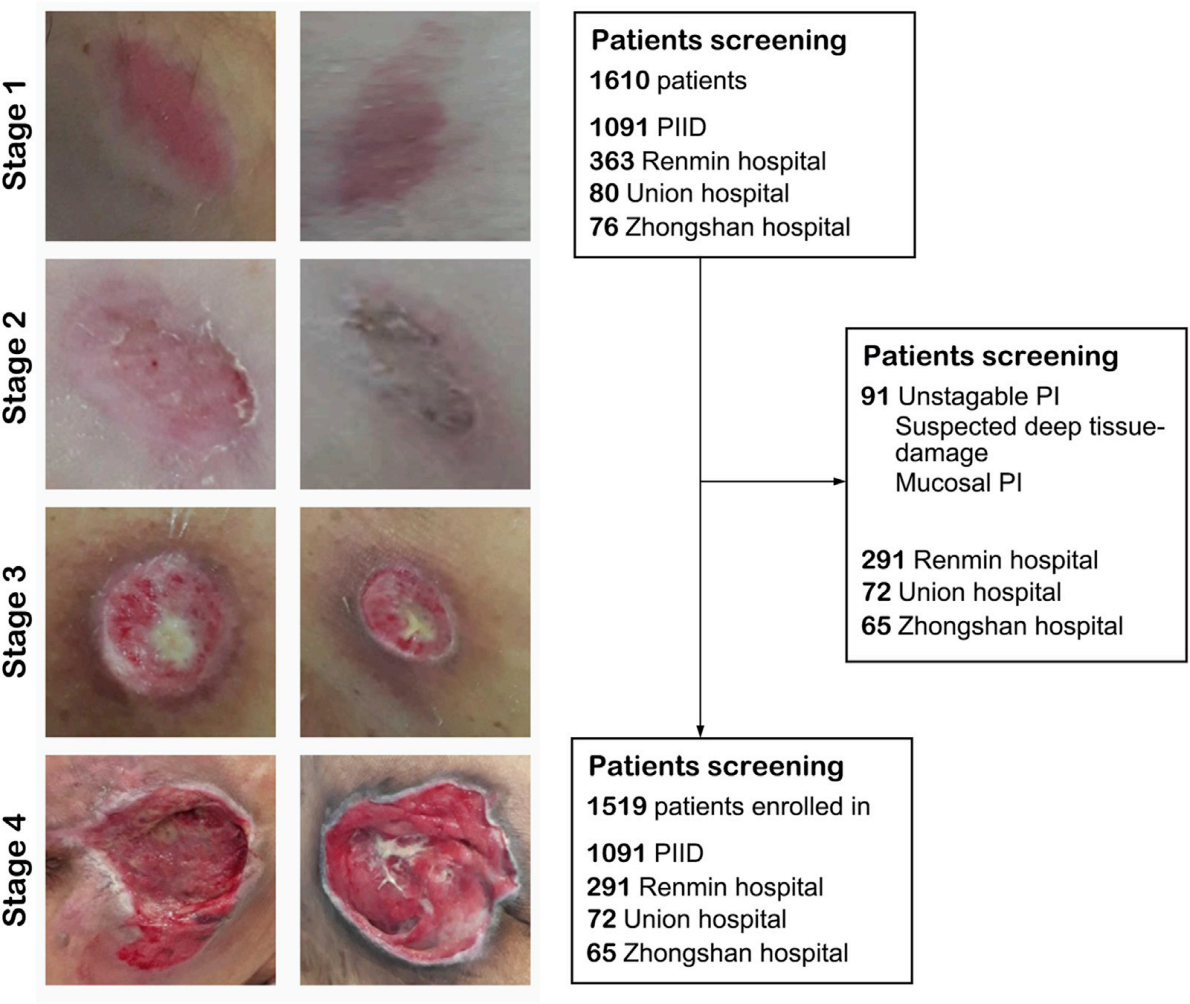
The flowchart of the present study. Left panel: Representative image of pressure injury from stage 1–4. Right panel: The inclusion and exclusion standard of our study. After screening, 1,091 images from PIID, 291 patients from Wuhan University Renmin Hospital, 72 patients from Union Hospital of Wuhan, and 65 patients from Zhongshan Hospital of Xiamen University were enrolled in.

We collected a total of 1,519 patients in this study, 1,216 as a train set and 303 as a validation set. The details of characteristics of patients in whu-PIID were presented in Table 2. Figure 2 showed the representative image of different stage of PI and the inclusion flowchart of patients among different cohorts.

**TABLE 2:** Characteristics of patients in whu-PIID.

	Whu-PIID set	
	N	100%
Sex		
Male	271	63.3
Female	155	36.2
Unknown	2	0.5
Age		
<50	192	44.9
>50	234	54.6
Unknown	2	0.5
Stage		
1	155	36.2
2	210	49.1
3	43	10.0
4	20	4.7

### 3.2 Algorithm performance

In this study, we used five state-of-the-art pre-trained CNN models to perform pressure ulcer classification tasks. Due to the requirement for detection speed in practical applications of pressure ulcer classification, we selected models with smaller sizes (such as small or tiny versions) or models with fewer stacked layers. The models we chose were ResNeXt50_32x4d (Xie et al., 2017), ConvNeXt-s (Liu et al., 2022), EfficientNetV2-s (Tan and Le, 2019; Tan and Le, 2021), DenseNet161 (Huang et al., 2016), and Swin-Transformer-tiny (Liu et al., 2021a). Additionally, we used resnext50_32x4d as the backbone and added our own designed wFPN (weighted Feature Pyramid Network) as a bottleneck for comparison experiment. We employed transfer learning by loading pre-trained weights from the ImageNet database into the backbone network and fine-tuning it on our own dataset to obtain the final training results. The sizes of the pre-trained weights of the models and the accuracy obtained on the validation set are shown in Table 3. Based on the evaluation metrics mentioned in Section 3.3, we found the overall accuracy, precision, recall, and F1-score of our networks were 0.815, 0.808, 0.816, and 0.811 respectively, indicating our improved network had better performance than other five networks (Table 4).

**TABLE 3:** The sizes of the pre-trained weights of each models and the accuracy obtained on the validation set.

Network	Model size (MB)	Accuracy (%)
ResNeXt50 + wFPN	95.7	81.5
ResNeXt50	95.7	79.5
EfficientNetV2-s	82.6	78.8
ConvNeXt-s	191	78.1
DenseNet161	110	76.2
Swin Transformer-tiny	109	75.5

**TABLE 4:** The overall accuracy, precision, recall, and F1-score of each models.

Network	Accuracy	Precision	Recall	F1-score
ResNeXt50 + wFPN	0.815	0.808	0.816	0.811
ResNeXt50	0.795	0.784	0.790	0.782
EfficientNetV2-s	0.788	0.781	0.775	0.776
ConvNeXt-s	0.781	0.771	0.777	0.773
DenseNet161	0.762	0.751	0.763	0.751
Swin Transformer-tiny	0.755	0.761	0.747	0.753

**FIGURE 3:**
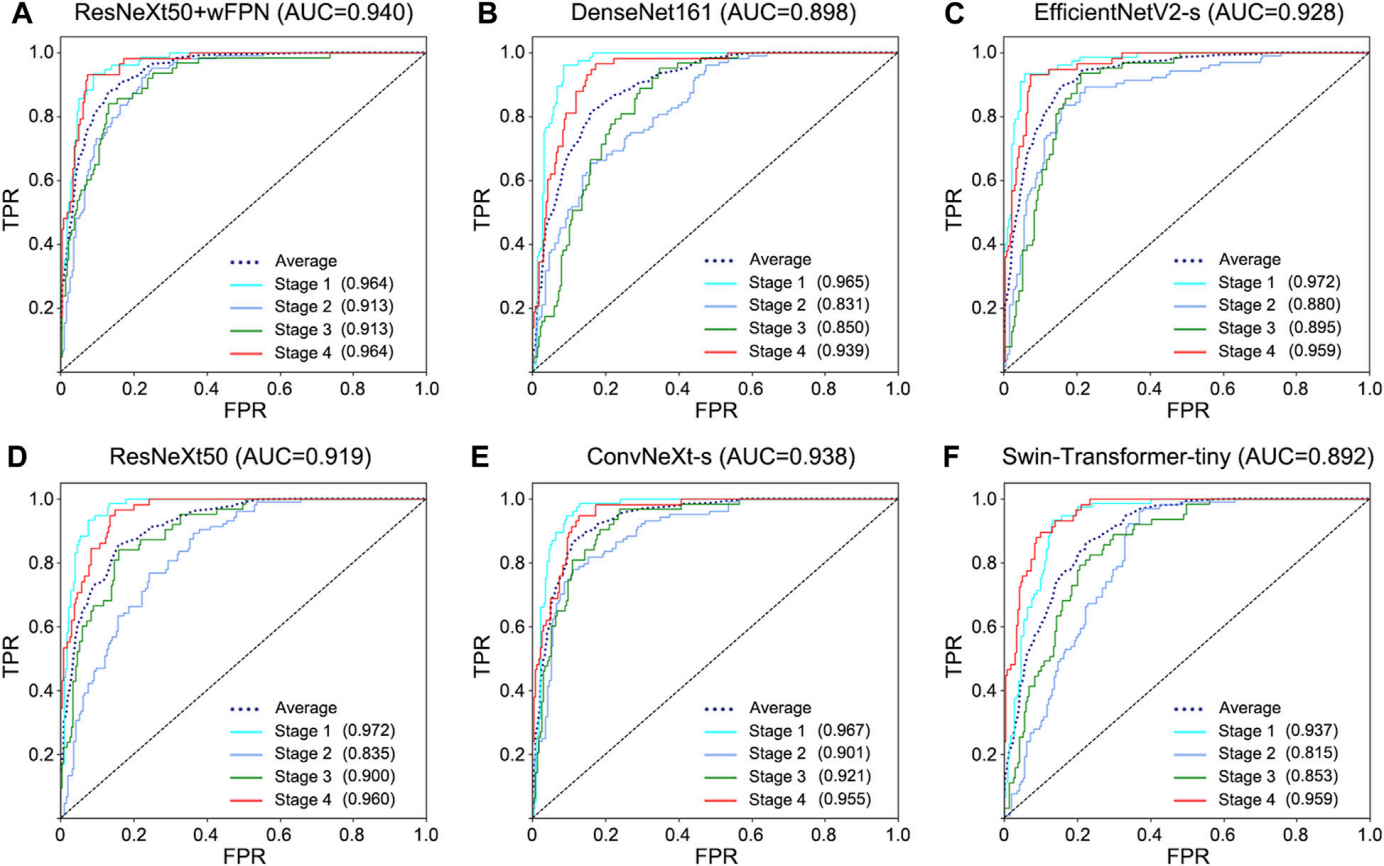
ROC curve for each network. **(A)** ResNeXt50+wFPN: the AUC for average, stage 1, stage 2, stage 3, and stage 4 were 0.940, 0.964, 0.913, 0.913, and 0.964, respectively. **(B)** DenseNet161: the AUC for average, stage 1, stage 2, stage 3, and stage 4 were 0.898, 0.965, 0.831, 0.850, and 0.939, respectively. **(C)** EfficientNetV2-s: the AUC for average, stage 1, stage 2, stage 3, and stage 4 were 0.928, 0.972, 0.880, 0.895, and 0.959, respectively. **(D)** ResNeXt50: the AUC for average, stage 1, stage 2, stage 3, and stage 4 were 0.919, 0.972, 0.835, 0.900, and 0.960, respectively. **(E)** ConvNeXt-s: the AUC for average, stage 1, stage 2, stage 3, and stage 4 were 0.938, 0.967, 0.901, 0.921, and 0.955, respectively. **(F)** Swin-Transformer-tiny: the AUC for average, stage 1, stage 2, stage 3, and stage 4 were 0.892, 0.937, 0.815, 0.853, and 0.959, respectively.

The receiver operating characteristic (ROC) curve can easily reveal the recognition capability of a classifier at a certain threshold, with the False Positive Rate (FPR) on the horizontal axis and the True Positive Rate (TPR) on the vertical axis. The closer the curve is to the upper-left corner, the higher the sensitivity and the lower the false positive rate, indicating better classification performance of the network. The area under curve (AUC) of ROC curve considers the classifier’s ability to classify positive and negative examples and provides a reasonable evaluation even in cases of imbalanced samples. We observed the highest AUC in our improved network (AUC = 0.940) among six networks (ResNeXt50: 0.919, ConvNeXt-s: 0.938, EfficientNetV2-s: 0.928, DenseNet161: 0.898, Swin-Transformer-tiny: 0.892) (Figure 3).

**FIGURE 4:**
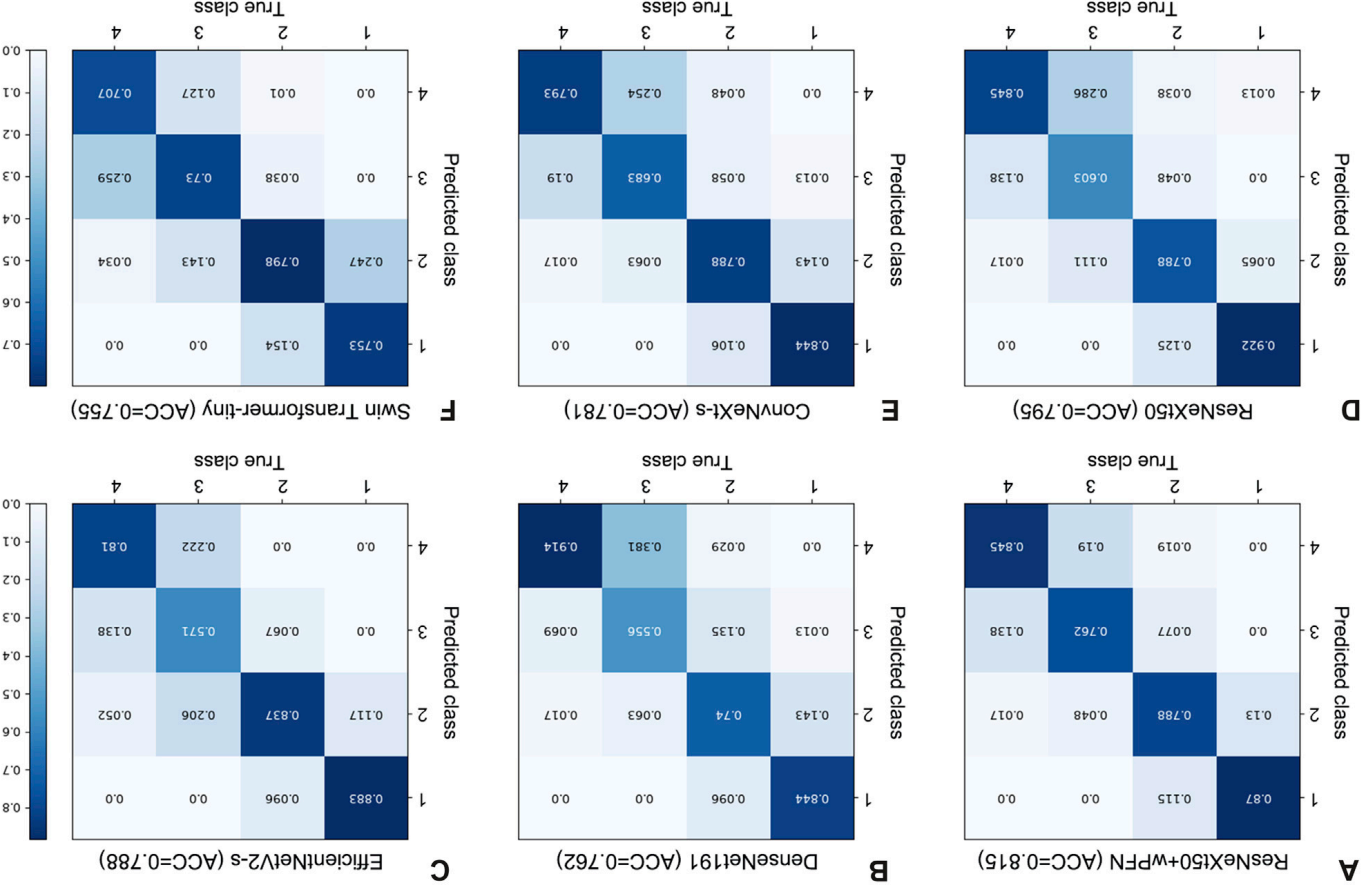
Confusion matrix for each network. **(A)** ResNeXt50+wFPN: the AUC for predicting stage 1, stage 2, stage 3, and stage 4 were 0.87, 0.788, 0.762, and 0.845, respectively. **(B)** DenseNet161: the AUC for predicting stage 1, stage 2, stage 3, and stage 4 were 0.844, 0.74, 0.556, and 0.914, respectively. **(C)** EfficientNetV2-s: the AUC for predicting stage 1, stage 2, stage 3, and stage 4 were 0.883, 0.837, 0.571, and 0.81, respectively. **(D)** ResNeXt50: the AUC for predicting stage 1, stage 2, stage 3, and stage 4 were 0.922, 0.788, 0.603, and 0.845, respectively. **(E)** ConvNeXt-s: the AUC for predicting stage 1, stage 2, stage 3, and stage 4 were 0.844, 0.788, 0.683, and 0.793, respectively. **(F)** Swin-Transformer-tiny: the AUC for predicting stage 1, stage 2, stage 3, and stage 4 were 0.753, 0.798, 0.73, and 0.707, respectively.

Based on the results obtained from each network’s validation, we plotted the normalized confusion matrix, with the true labels on the horizontal axis and the predicted labels on the vertical axis (Figure 4). From the graph, we can see that ResNeXt50, EfficientNetV2-s, ConvNeXt-s, and DenseNet161 have a higher misdiagnosis rate for stage 3 (ResNeXt50: 60.3%, EfficientNetV2-s: 57.1%, ConvNeXt-s: 68.3%, and DenseNet161: 55.6%), which is the main factor limiting their overall accuracy. Swin Transformer-tiny shows relatively average performance across all categories, but the accuracy for all four stages is generally low in the 70%–80% range. On the other hand, ResNeXt50 + wFPN achieves a classification accuracy of over 75% for each stage, with particularly outstanding performance in stage 1 (87%) and stage 4 (84.5%), which contributes to its overall superior performance compared to the other five networks.

## 4 Discussion

To date, PI has become a global health problem. The key to the treatment of PI is early diagnosis, classification and prevention, which requires a concerted effort both inside and outside the hospital. Compared with HAPI, more serious PI often occurs around us. Early identification of them can effectively block the disease process and improve the survival of patients. Herein, we developed a novel classification method for PI based on deep learning in a multi-center cohorts. Our model increased the overall accuracy from 75% to 81.5%, comparing with Ay et al. work (Ay et al., 2022). We also increased the accuracy for predicting stage 3 PI from 60.3% to 76.2% through adding wFPN. This method holds promise for precise diagnosis and classification of PI to achieve homogenous management within hospitals and provide medical advice to post-charged patients, and will greatly improve the life quality of patients and save medical resources.

Actually, we have noticed a number of groups that have undertaken some inspiring work in the early diagnosis field of PI. For instance, Cai et al. (Jiang et al., 2020; Cai et al., 2021; Jiang et al., 2022; Chen et al., 2023) developed several machine-learning based methods and highlighted the application of infrared thermography detection in PI diagnosis. Song et al. (Song et al., 2021) also established a data-driven, generalizable pressure injury prediction model in a relatively large cohort. These works strongly promote the development of PI prediction. However, compared with early diagnosis, precise classification of PI faced with more challenges. Different from the large number of quantified clinical features as parameters for early diagnosis, PI classification depends more on the learning and recognition of image features. Therefore, there is a lack of related research. However, the importance of PI precise classification is as important as early diagnosis. In particular, higher degree PI commonly occurs in patients out of hospital, who lack of professional medical knowledge. Thus, development of an automatic classification model hold promise in inhibiting PI progression and improving patients’ life quality.

Compared with machine-learning methods, neural network based deep-learning showed greater capability in PI classification. To date, Ay et al. (Ay et al., 2022) constructed PI classification network through deep learning. However, their deep-learning based model can only achieve an accuracy of ∼75%. We notice that their model showed remarkably lower accuracy in distinguishing stage 3 from others, which is also challenging in clinical practice. In fact, the information provided by flat images is limited, and they cannot provide information of three-dimensional level such as temperature and depth. Adding methods such as skin temperature detection will make PI prediction more complex and difficult to perform at home, which is not in line with our original intention. Therefore, we hope to get more information from the images to make up for the lack of three-dimensional information. Feature fusion is the process of taking features extracted from an image and combining them into one feature that is more discriminating than the input features (Rahaman et al., 2021; Chen et al., 2022; Zhang et al., 2023). In the use of CNNs for extracting image features, it is generally believed that the early layers of the network can capture low-level features of the image, while deeper layers can get high-level features. Low-level features have higher resolution and contain more positional and detailed information, but they have lower semantic meaning and more noise due to fewer convolutions. High-level features, on the other hand, have stronger semantic information but lower resolution and less ability to perceive details. The fusion of multi-scale features aims to better utilize the different level of features and model them together. Early approaches to feature fusion include feature concatenation, feature summation (Lin et al., 2017), skip connections (Liu et al., 2018a), deconvolution (Fu et al., 2017), and multi-scale model (Li and Zhou, 2017). Thereafter, various complex feature fusion networks have emerged. TFN and LFN (Zadeh et al., 2017; Liu et al., 2018b) perform feature fusion by taking the outer product of different features, but they are prone to the problem of dimension explosion, and the fusion effect is not ideal. Sahu et al. (Sahu and Vechtomova, 2019) propose using autoencoder and generating adversarial networks (GAN) for fusion, but this approach reduces the dimensionality of the original features, leading to the loss of some features. Chaib et al. (Chaib et al., 2017) introduce discriminant correlation analysis (DCA) into canonical correlation analysis (CCA) to maximize the correlation between corresponding features in two feature sets while maximizing the differences between different classes. This strategy has advantage of fusing features by calculating the transformations between two input features, but ignores the relationships between class structures in the dataset. Based on the idea of weighted summation, a lot of study introduce attention mechanisms (Vielzeuf et al., 2018; Liu et al., 2019; Wu et al., 2021), self-attention mechanisms (Liu et al., 2021b), and gating mechanisms (Pu et al., 2020) into feature layers to reflect the different contributions of feature layers with different input resolutions to the final result. In view of the inconsistent quality, complex information, and obvious individual differences of images, our own designed wFPN showed significantly better performance than previous deep-learning models. Through our models, we first increase the overall accuracy from 75% to 81.5%, compared with Ay et al. Work (Ay et al., 2022). Then, we increased the accuracy for predicting stage 3 PI from 60.3% to 76.2% through adding wFPN. After further validation, our developed PI classification model holds promise in achieving clinical translation.

The great clinical application is another strength of our study. We collected 1,519 images from multi-center cohorts, including different hospitals, race, and shooting conditions. Through this database, our model provided a relatively accurate and objective classification results, which is helpful for unexperienced nurses to determine the condition of patients. This is also conducive to the homogeneous management of patients. Besides, it provides a convenient and quick way for family members to learn about the condition of PI in patients. As well known, severe PI are more likely to occur outside the hospital because family members or caregivers mostly lack professional medical knowledge and training. Our study provides a simple and convenient means to accurately judge and stage pressure ulcers in patients with only an ordinary mobile phone. When the PI is in the early stage, it can prompt the patient to early prevention and treatment; when the situation of PI is more serious, it can prompt patients to be hospitalized in time. This can greatly save medical resources and relieve the pressure of hospitalization. More importantly, this model has an extensive application prospect. Although PI affects 3 million people each year, the cases that really happened in large teaching hospitals or tertiary hospitals are actually very limited, which restricts us from obtaining more images to further optimize network. Our model pays more attention to the use of patients outside the hospital, which means that we can receive more feedback and images to further optimize our software.

However, there are still some limitations. First, we could not quantify the depth of PI, leading to confusion between stage 3 PI and other stages. Second, although we collected 1,519 images from multi-center cohorts as train set, the patients in validation set remained insufficient. This is also because it is not easy to obtain PI images from hospitals after more attpention has been paid to the prevention of PI. Therefore, future work needs to focus on: 1) obtaining images from patients out of hospital; 2) expanding the clinical sample and combining with other technical means, including infrared thermography detection, to provide more parameters for deep-learning network to achieve more accurate classification of PI.

## 5 Conclusion

For the first time, we combined CNN and wFPN network to construct the accurate classification model of PI in the multi-center dataset of more than 1,500 samples. Compared with other current networks, our model showed great capability in PI classification. Upon further validation, our study will pave the path to the clinical application of our network in PI management.

## Data Availability

The raw data supporting the conclusion of this article will be made available by the authors, without undue reservation.
